# KLF13-mediated CES2 upregulation via p300-dependent acetylation sensitizes gastric cancer cells to irinotecan

**DOI:** 10.1016/j.isci.2025.114199

**Published:** 2025-11-22

**Authors:** Hai-bin Zhang, Ren-hao Hu, Ke-hui Zhang, Xi-mao Cui, Shun Zhang

**Affiliations:** 1 Department of Gastrointestinal Surgery, Shanghai East Hospital, School of Medicine, Tongji University, Shanghai, China; 2 Center of Digestive Endoscopy, Shanghai East Hospital, School of Medicine, Tongji University, Shanghai, China

**Keywords:** biological sciences, molecular medicine, cancer systems biology, cancer

## Abstract

Irinotecan (CPT-11) is a key chemotherapeutic agent for gastric cancer (GC), but its efficacy is limited by variable patient responses. The activation of CPT-11 to its active form, SN-38, depends on the enzyme carboxylesterase 2 (CES2), yet the regulation of CES2 in GC is poorly understood. Here, we identify Krüppel-like factor 13 (KLF13) as a direct transcriptional activator of CES2. We show that KLF13 expression correlates with CES2 levels and treatment response in GC patients. Mechanistically, KLF13 binds to specific elements in the CES2 promoter to drive its expression. This function of KLF13 is critically dependent on its acetylation by the co-activator p300, as demonstrated by site-directed mutagenesis and p300 inhibition. Functionally, the KLF13-CES2 axis dictates cellular sensitivity to CPT-11. Our findings unveil a p300-KLF13-CES2 signaling pathway that governs irinotecan sensitivity, providing potential predictive biomarkers and therapeutic targets for GC.

## Highlights

• KLF13 expression is associated with CPT-11 response in gastric cancer patients
• KLF13 directly binds to and transcriptionally activates the CES2 promoter
• p300 acetylates KLF13, a modification critical for its function
• The p300-KLF13-CES2 axis is a novel determinant of irinotecan sensitivity

## Graphical abstract

**Figure:**
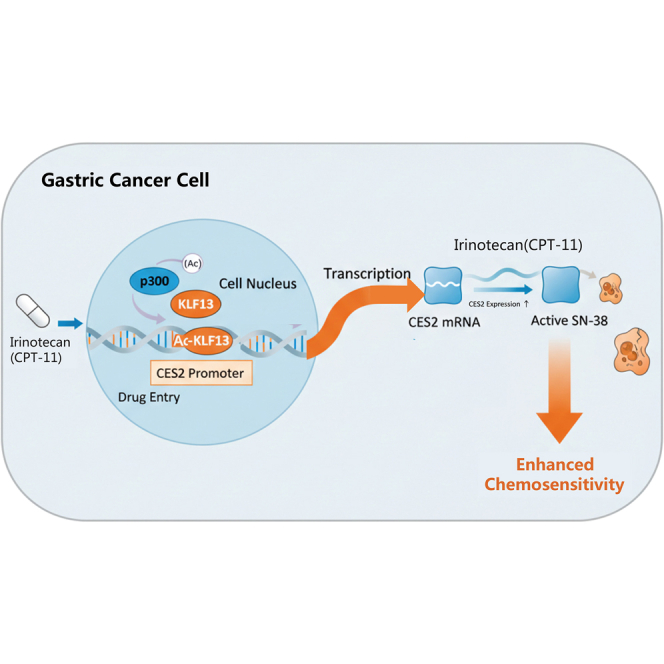

## Introduction

Gastric cancer (GC) remains a major global health challenge, characterized by a high mortality rate and limited therapeutic options, particularly for patients with advanced or metastatic disease.^1^^,^^2^ Irinotecan (CPT-11), a topoisomerase I inhibitor, is a key agent in the therapeutic arsenal, especially as a second-line or later-line salvage therapy for advanced GC.^3^^,^^4^^,^^5^ However, its clinical utility is severely hampered by highly variable patient responses. This stark variability underscores a critical unmet need for predictive biomarkers. The clinical utility of irinotecan is further complicated by a therapeutic paradox centered on its bioactivation enzyme, carboxylesterase 2 (CES2).^6^^,^^7^ High intratumoral CES2 expression is desirable for therapeutic efficacy, a concept strongly supported by survival data in pancreatic cancer.^8^ However, high intestinal CES2 activity is a primary culprit behind severe, dose-limiting diarrhea.^9^ This dual role highlights the importance of understanding the tumor-specific transcriptional regulation of the CES2 gene.

To identify potential transcriptional regulators of CES2 in GC, we initiated our study by screening the expression of several Krüppel-like factor (KLF) family members, known for their multifaceted roles in tumorigenesis,^10^^,^^11^ in our cohort of clinical GC specimens. This preliminary screen revealed significant positive correlations between CES2 mRNA levels and those of both KLF4 and KLF13 (data not shown). The specific role of KLF13 is context dependent and complex; it can act as a tumor suppressor in GC via the β-catenin pathway,^12^ whereas a recent study identified KLF13 as a pro-tumorigenic factor in esophageal cancer.^13^ This complexity, particularly in the context of drug metabolism, prompted us to prioritize KLF13 for in-depth investigation. Given that its role in regulating drug metabolism pathways was entirely unknown, we prioritized KLF13 for in-depth investigation to determine if it represents a node connecting tumor suppression to chemosensitivity.

The activity of transcription factors is often fine-tuned by post-translational modifications (PTMs) like acetylation, catalyzed by histone acetyltransferases (HATs) such as p300/CBP.^14^^,^^15^ The functional interplay between KLF13 and p300/CBP via acetylation has been previously described,^16^ leading to our central hypothesis: that the tumor-specific expression of CES2 is controlled by a multi-layered cascade, initiated by p300-mediated acetylation of KLF13, which in turn transcriptionally activates CES2. Dissecting this pathway could provide not only a powerful predictive biomarker but also uncover therapeutic strategies to selectively enhance irinotecan’s efficacy within the tumor.

## Results

### KLF13 expression positively correlates with CES2 in gastric cancer and is associated with CPT-11 treatment response

**Figure 1:**
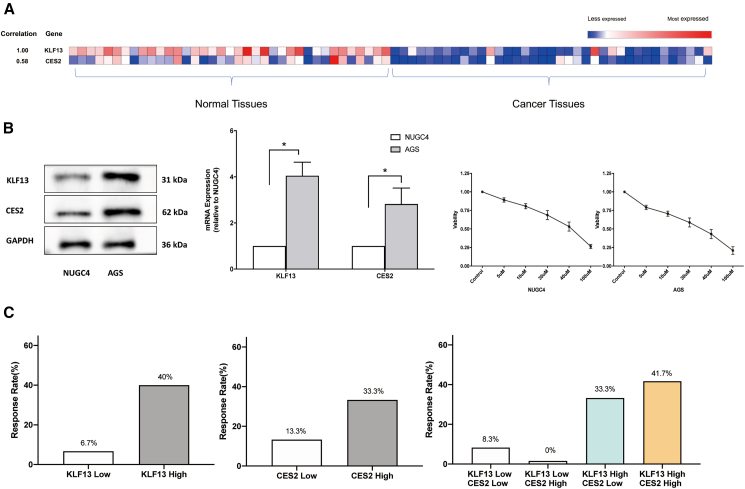
KLF13 expression correlates with CES2 and CPT-11 sensitivity in GC (A) Heatmap depicting the relative mRNA expression of KLF13 and CES2 in 37 pairs of GC tissues and adjacent normal tissues. The Pearson correlation coefficient (r) and its significance (*p* value) are shown on the left (r = 0.58, *p* < 0.001). (B) Baseline characterization of NUGC4 and AGS GC cell lines. (Left) western blot (WB) analysis of endogenous KLF13 and CES2 proteins. (Middle) qPCR analysis of KLF13 and CES2 mRNA. Expression in AGS cells is relative to NUGC4 (set to 1). (Right) Cell viability curves after 48 h CPT-11 treatment. (C) Correlation between KLF13/CES2 mRNA expression and ORR in 30 GC patients receiving CPT-11-based second-line chemotherapy. Patients were stratified into high/low groups by the median. ORR was compared using Fisher’s exact test. Data in (B) are mean ± SD (*n* = 3). ∗*p* < 0.05.

To investigate the potential clinical relevance of KLF13 and CES2 in GC, we first analyzed their expression in a cohort of patient-derived specimens. A heatmap analysis of our patient cohort (*n* = 37) revealed a statistically significant positive correlation between the mRNA levels of KLF13 and CES2 (Pearson correlation coefficient, r = 0.58, *p* < 0.001; Figure 1A). Notably, the expression of both genes generally appeared to be downregulated in cancer tissues compared to adjacent non-tumor tissues. This finding was further supported by analysis of The Cancer Genome Atlas (TCGA) Stomach Adenocarcinoma cohort, which also showed downregulation of both CES1 and CES2 in tumor tissues, with CES2 being the predominantly expressed isoform (Figures S1A and S1B).

We next characterized the baseline expression and drug sensitivity in two human GC cell lines, NUGC4 and AGS. Consistent with the TCGA data, qPCR analysis confirmed that CES2 was the major CES isoform expressed in both cell lines (Figure S1C). AGS cells, which expressed higher endogenous levels of both KLF13 and CES2 protein and mRNA, were markedly more sensitive to the chemotherapeutic drug CPT-11 (irinotecan) than NUGC4 cells, which exhibited lower KLF13 and CES2 expression (Figure 1B).

To translate these findings into a clinical context, we analyzed the treatment response in a cohort of 30 advanced GC patients who had received CPT-11-based second-line chemotherapy. Patients were stratified into high- and low-expression groups based on the median tumoral mRNA levels. A strong trend emerged where patients with high KLF13 expression exhibited a substantially higher objective response rate (ORR) compared to those with low expression (40.0% vs. 6.7%; *p* = 0.0818, Fisher’s exact test; Figure 1C, left panel). A similar trend, although not statistically significant, was observed for CES2 expression (33.3% vs. 13.3%; Figure 1C, middle panel). Strikingly, a combined analysis showed that patients with high expression of both KLF13 and CES2 achieved the highest ORR (41.7%), whereas patients with low KLF13 expression showed a minimal response, regardless of CES2 levels, suggesting that KLF13 expression might be a dominant factor associated with treatment response (Figure 1C, right panel). Collectively, these clinical and cellular data establish a strong link between KLF13, CES2, and CPT-11 efficacy, prompting further mechanistic investigation.

### KLF13 directly regulates CES2 transcription by binding to its promoter

**Figure 2:**
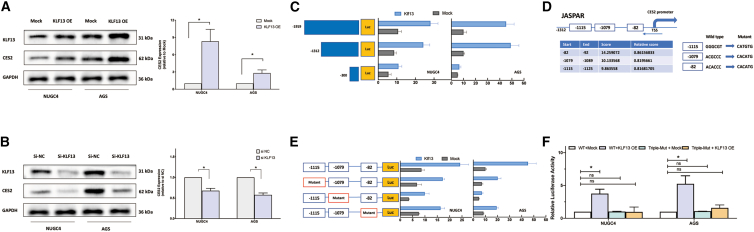
KLF13 directly regulates CES2 transcription (A and B) WB (left) and qPCR (right) analysis of CES2 expression in NUGC4 and AGS cells 48 h after (A) KLF13 overexpression (OE) or (B) KLF13 knockdown (si-KLF13). See also Figure S1 for KLF13 expression efficiency. (C) Luciferase activity of serially deleted CES2 promoter constructs co-transfected with KLF13 OE or Mock vector in NUGC4 and AGS cells. (D) Schematic of the CES2 promoter (−1,312 bp) showing predicted KLF13-binding sites from JASPAR. (E) Luciferase activity of the −1,312-bp promoter with individual site mutations. (F) Luciferase activity of the −1,312-bp promoter with all three sites mutated (Triple-Mut). Data are mean ± SD (*n* = 3). ns, not significant. ∗*p* < 0.05.

Given the strong correlation, we next sought to determine if KLF13 directly regulates CES2 expression. In both NUGC4 and AGS cells, ectopic overexpression of KLF13 resulted in a marked upregulation of CES2 at both the protein and mRNA levels (Figure 2A). The efficiency of overexpression was confirmed by qPCR (Figure S1E). Conversely, small interfering RNA -mediated knockdown of KLF13, confirmed at the mRNA level (Figure S1F), led to a significant reduction in CES2 expression (Figure 2B). Importantly, KLF13 did not regulate the expression of the CES1 isoform, indicating a specific regulatory effect on CES2 (Figure S1D).

To elucidate the underlying transcriptional mechanism, we performed a series of luciferase reporter assays. A deletion analysis of the CES2 promoter revealed that KLF13 overexpression significantly activated the −1,919- and −1,312-bp promoter constructs, while this activation was markedly attenuated in the −300-bp construct, suggesting that key KLF13-responsive elements are located upstream of the −300-bp region (Figure 2C). Using the JASPAR database, we predicted three potential KLF13 binding sites within the −1,312-bp region, located at positions −1,115, −1,079, and −82 relative to the transcription start site (Figure 2D). To verify the functionality of these sites, we performed site-directed mutagenesis. Individual mutation of each of the three predicted sites significantly blunted the KLF13-induced activation of the CES2 promoter (Figure 2E). Furthermore, a triple-mutant construct, with all three sites simultaneously mutated, showed a complete abrogation of KLF13-mediated promoter activation (Figure 2F). These findings provide strong evidence that KLF13 directly binds to these specific *cis*-regulatory elements to activate CES2 promoter activity.

### The KLF13-CES2 axis is a functional determinant of CPT-11 chemosensitivity

**Figure 3:**
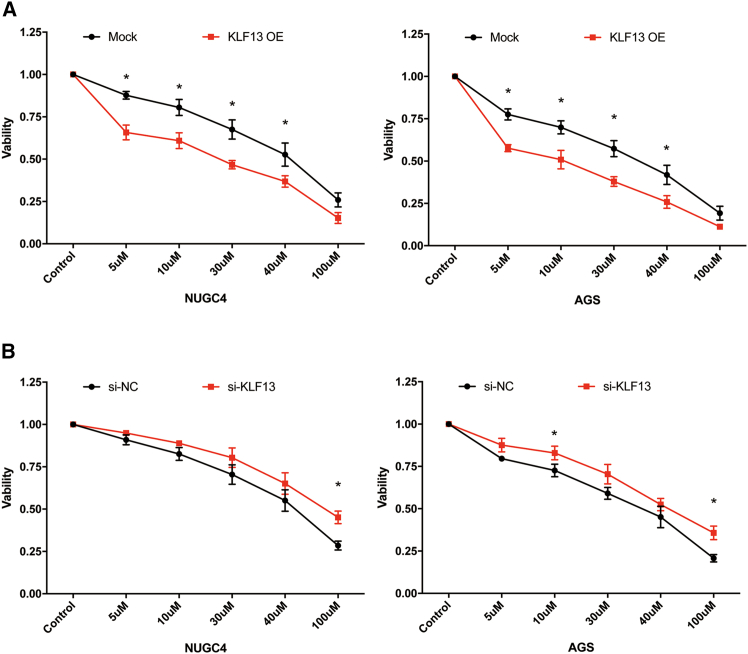
KLF13 expression dictates cellular sensitivity to CPT-11 Cell viability curves of NUGC4 and AGS cells treated with CPT-11 for 48 h following (A) KLF13 overexpression or (B) KLF13 knockdown. Data are mean ± SD (*n* = 3). ∗*p* < 0.05 indicates significant difference between the two groups at the indicated concentration, determined by Student’s *t* test.

Next, we investigated whether the KLF13-mediated regulation of CES2 translates into altered chemosensitivity. As determined by XTT assays, overexpression of KLF13 significantly increased the sensitivity of both NUGC4 and AGS cells to CPT-11, as evidenced by a downward shift in the viability curves (Figure 3A). In contrast, knockdown of KLF13 rendered the cells more resistant to CPT-11 treatment (Figure 3B). These results establish a direct functional link between the KLF13-CES2 regulatory axis and cellular response to CPT-11.

### p300 acetyltransferase activity is essential for KLF13-mediated regulation of CES2

**Figure 4:**
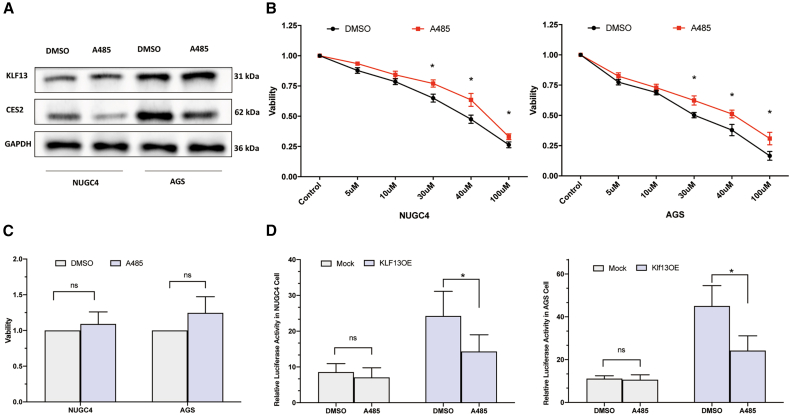
p300 acetyltransferase activity is essential for the KLF13-CES2 axis (A) WB analysis of KLF13 and CES2 expression in cells treated with DMSO or p300 inhibitor A485 (3 μM) for 24 h. (B) Cell viability curves of cells pre-treated with DMSO or A485 for 6 h, followed by 48-h CPT-11 treatment. (C) Viability of cells treated with DMSO or A485 alone for 48 h. (D) Luciferase activity of the CES2 −1,312-bp promoter in cells co-transfected with KLF13 OE (or Mock) and treated with DMSO or A485. Data are mean ± SD (*n* = 3). ns, not significant; ∗*p* < 0.05.

Given that KLF13 activity can be modulated by co-activators, we explored the role of the acetyltransferase p300. Treatment with A485, a selective p300/CBP inhibitor, significantly reduced CES2 protein expression without affecting KLF13 protein levels in both cell lines (Figure 4A). Functionally, pre-treatment with A485 antagonized the cytotoxic effects of CPT-11, resulting in increased cell viability and resistance (Figure 4B). Importantly, A485 alone did not exhibit significant cytotoxicity at the concentration used (Figure 4C). Furthermore, in luciferase reporter assays, A485 treatment markedly suppressed the ability of KLF13 to activate the CES2 promoter (Figure 4D). These data collectively indicate that the acetyltransferase activity of p300 is essential for KLF13-mediated regulation of CES2.

### p300-mediated acetylation of KLF13 is the critical molecular switch for its transcriptional activity

**Figure 5:**
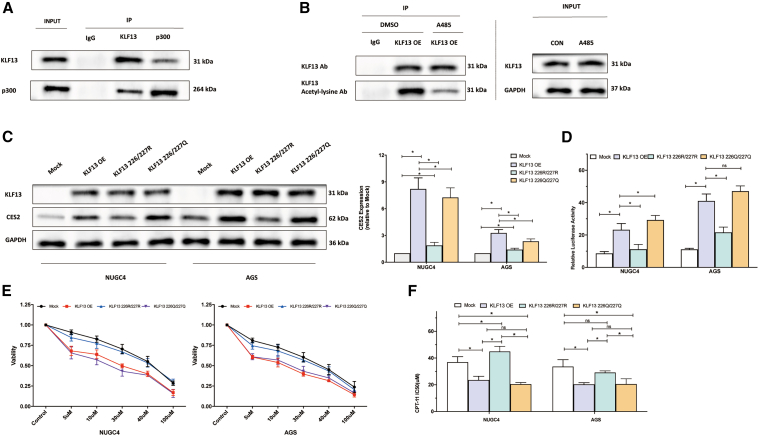
p300-mediated acetylation of KLF13 is the functional molecular switch (A) CoIP of endogenous KLF13 and p300 from AGS cell lysates. (B) IP-WB analysis of KLF13 acetylation in AGS cells treated with DMSO or A485. (C) WB and qPCR analysis of CES2 expression in cells overexpressing KLF13-WT, K226/227R, or K226/227Q mutants. See also Figure S2 for mutant expression efficiency. (D) Luciferase activity of the CES2 −1,312-bp promoter in cells co-transfected with KLF13-WT or mutants. (E and F) (E) Cell viability curves and (F) corresponding IC50 values for CPT-11 in cells overexpressing KLF13-WT or mutants. Data are mean ± SD (*n* = 3). ns, not significant; ∗*p* < 0.05.

To define the precise mechanism of p300’s involvement, we first confirmed a physical interaction between KLF13 and p300 in GC cells via co-immunoprecipitation (coIP) assays (Figure 5A). We then assessed KLF13 acetylation via immunoprecipitation-Western Blot (IP-WB) analysis. The results showed that KLF13 is endogenously acetylated and that this modification was substantially diminished upon treatment with the p300 inhibitor A485, confirming p300-dependent KLF13 acetylation (Figure 5B).

Finally, to prove the functional significance of this PTM, we performed functional studies using KLF13 site-directed mutants at the predicted acetylation sites K226/227: an acetylation-deficient mutant (K226/227R) and an acetylation-mimicking mutant (K226/227Q). While all KLF13 constructs were expressed at comparable protein and mRNA levels (Figure 5C, top and middle panels; Figure S2), their ability to regulate CES2 and CPT-11 sensitivity differed dramatically. Compared to wild-type KLF13 (WT), the K226/227R mutant showed a significantly impaired ability to upregulate CES2 protein and mRNA levels (Figure 5C), activate the CES2 promoter (Figure 5D), and sensitize cells to CPT-11, as shown by both cell viability curves and a significantly higher IC50 value (Figures 5E and 5F). In contrast, the K226/227Q mutant largely phenocopied the effects of KLF13-WT in all these assays. These results unequivocally demonstrate that p300-mediated acetylation at specific lysine residues is the crucial molecular switch that enables KLF13’s transcriptional activity and its subsequent function in regulating CES2 expression and CPT-11 sensitivity in GC.

## Discussion

The unpredictable efficacy of irinotecan in advanced GC poses a significant clinical challenge. The present study addresses this challenge by uncovering a multi-tiered regulatory axis—p300-KLF13-CES2—that governs cellular sensitivity to CPT-11. Our work provides a comprehensive, mechanistically linked narrative that begins with clinical observations and culminates in the functional validation of a specific PTM. We demonstrate that the transcription factor KLF13 is a direct transcriptional activator of the critical drug-metabolizing enzyme CES2. More profoundly, we reveal that this regulatory function of KLF13 is itself controlled by p300-mediated acetylation, thus establishing a direct link between a key signaling co-activator and the machinery of chemotherapy metabolism.

Our study was grounded in clinical reality, where we observed a positive correlation between KLF13 and CES2 mRNA expression in GC tissues and a strong trend associating their higher expression with better clinical response to CPT-11. Although our clinical analysis did not reach statistical significance (*p* > 0.05), likely due to the limited size of our retrospective cohort (*n* = 30), the magnitude of the observed effect is clinically suggestive and aligns powerfully with our *in vitro* findings. This observation is reminiscent of the pivotal study in pancreatic cancer by Capello et al.,^8^ where high tumoral CES2 expression was a strong independent predictor of longer survival. An intriguing observation from our clinical cohort, however, was that KLF13 expression appeared to be a more dominant predictor of ORR than CES2 expression alone. While high CES2 levels are mechanistically required for irinotecan activation, our data showed that patients with a KLF13^low^CES2^high^ expression signature still exhibited a poor response (0% ORR; Figure 1C). This initially seems counterintuitive. However, this finding may suggest a more complex role for KLF13 beyond simply being a transcriptional activator of CES2. It is plausible that KLF13, as a tumor suppressor, orchestrates a broader cellular state that is permissive for chemotherapy-induced apoptosis, for instance, by regulating other key pathways like β-catenin.^12^ In this model, high CES2 expression alone might be insufficient to overcome a generally resistant cellular phenotype driven by the loss of KLF13. Therefore, KLF13 expression may reflect both the capacity for drug activation (via CES2) and the intrinsic cellular readiness to undergo apoptosis, making it a potentially more robust biomarker than CES2 alone.

However, the role of CES2 in irinotecan therapy represents a classic “significant therapeutic challenge,” which lies at the heart of the therapeutic challenge. While our data and others underscore the benefit of high tumoral CES2 for drug efficacy,^6^ compelling evidence from Yu et al.^9^ demonstrates that high intestinal CES2 activity is a primary culprit behind the severe, dose-limiting diarrhea. This creates a critical therapeutic dilemma: systemically increasing irinotecan dose inevitably exacerbates intestinal toxicity. Therefore, our findings lead to a pivotal conclusion: enhancing the antitumor efficacy of irinotecan hinges less on elevating its systemic blood concentration and more on increasing its localized bioactivation within the tumor itself. The discovery that KLF13 is a key transcriptional regulator of tumoral CES2 expression becomes particularly significant in this context.

The mechanistic cornerstone of our study is the definitive demonstration of KLF13 as a direct transcriptional regulator of CES2. This finding adds a layer to the known transcriptional control of CES2, which has been previously linked to tumor suppressors like p53^17^ and metabolic regulators such as HNF4α.^18^ The implication of our work is further amplified by the discovery of the “acetyl-switch” mechanism. Our experiments demonstrate that p300 physically interacts with KLF13 and mediates its acetylation at K226/227. This finding is consistent with and significantly extends the seminal work by Song et al.,^16^ who first identified these residues as key acetylation sites for KLF13. Our study now assigns a specific and critical functional consequence to this acetylation event: the regulation of CES2 and, consequently, irinotecan sensitivity. This finding places KLF13 within a well-established paradigm where HATs like p300/CBP act as master regulators of transcription factor function.^15^^,^^19^

The elucidation of the p300-KLF13-CES2 pathway has broader implications. It provides a clear mechanistic basis for why enhancing intratumoral drug activation is a superior therapeutic strategy. Moreover, our findings contribute to an emerging paradigm where KLF family members act as “chemosensitizing factors.” The synergy we observed is conceptually parallel to the work of Tung et al.,^20^ who demonstrated a powerful synergy between KLF9 and histone deacetylases (HDAC) inhibitors, and other studies linking KLF4 and KLF17 to cisplatin and 5-FU sensitivity, respectively.^21^^,^^22^ This suggests that exploring the KLF13 status in tumors might not only predict response to irinotecan but also could identify patient populations that might benefit from combination strategies with epigenetic modulators, given the complex relationship between HATs and HDACs on KLF activity.^23^

In conclusion, our study unveils a complete signaling pathway critical for irinotecan sensitivity in GC. The p300-mediated acetylation of KLF13 enhances its ability to directly activate CES2 transcription, a mechanism distinct from other known resistance pathways such as drug efflux or altered DNA repair.^24^ Crucially, by linking a tumor-suppressive transcription factor to a key drug activation enzyme, our findings champion a perspective on optimizing irinotecan therapy: focusing on tumor-specific activation rather than systemic exposure. These findings strongly support the potential of a KLF13/CES2 expression signature as a predictive biomarker to guide the use of CPT-11 in a personalized manner, a critical need highlighted by clinical trials demonstrating variable responses in the salvage setting.^4^^,^^5^ Future prospective studies in larger patient cohorts are imperative to validate this biomarker signature. Moreover, the components of this pathway, particularly the KLF13-p300 axis, represent therapeutic targets for modulating irinotecan resistance and improving outcomes for patients with advanced GC.

### Limitations of the study

This study has several limitations. First, our clinical analysis was based on a retrospective cohort with a limited sample size (*n* = 30), which may have insufficient statistical power to detect all significant associations. The strong trend observed between KLF13 expression and treatment response, for instance, requires validation in larger, prospective clinical trials. Second, our mechanistic studies were performed entirely *in vitro* using established cell lines. While these models provide valuable insights, they do not fully recapitulate the complexity of the tumor microenvironment. Future studies using *in vivo* models, such as patient-derived xenografts, would be essential to confirm our findings and evaluate the therapeutic potential of targeting the KLF13-p300 axis. Finally, while we identified K226/227 as critical acetylation sites, we cannot exclude the possibility that other PTMs on KLF13 may also contribute to its regulatory function.

## Resource availability

### Lead contact

Further information and requests for resources and reagents should be directed to and will be fulfilled by the lead contact, Shun Zhang (v2zs@hotmail.com, zhangshun@tongji.edu.cn).

### Materials availability

All unique reagents generated in this study, including plasmid constructs, are available from the lead contact upon reasonable request.

### Data and code availability

• All data reported in this paper will be shared by the lead contact upon reasonable request.
• This paper does not report original code.
• Any additional information required to reanalyze the data reported in this paper is available from the lead contact upon request.

## Acknowledgments

The study is funded by Pudong New Area Science and 10.13039/100006180Technology Development Fund for Livelihood Research Special Project (no. PKJ2023-Y38).

## Author contributions

S.Z., conceptualization, methodology, validation, formal analysis, investigation, writing – original draft, supervision, project administration, and funding acquisition. H.-b.Z., validation, formal analysis, investigation, writing – review and editing, and visualization. R.-h.H., validation, formal analysis, investigation, writing – review and editing, and visualization; K.-h.Z., validation, investigation, and writing – review and editing. P.L., resources, data curation, and writing – review and editing. X.-m.C., methodology, resources, and writing – review and editing.

## Declaration of interests

The other authors declare no conflict of interest.

## STAR★Methods

### Key resources table

**Table:** 

REAGENT or RESOURCE	SOURCE	IDENTIFIER
**Antibodies**		

Mouse polyclonal KLF13 antibody	Proteintech	Cat# 18352-1-AP RRID: AB_2132399
Rabbit polyclonal CES2 antibody	Proteintech	Cat# 15378-1-AP; RRID: AB_2077501
Rabbit polyclonal p300 antibody	Proteintech	Cat# 20695-1-AP; RRID: AB_3085614
Rabbit monoclonal acetyl-Lysine antibody	Abcam	Cat# Ab190479; RRID: AB_446436
Mouse monoclonal GAPDH antibody	Proteintech	Cat# 60004-1-Ig; RRID: AB_2107436
Mouse monoclonal DYKDDDDK tag antibody (FLAG)	Proteintech	Cat# 66008-4-Ig; RRID: AB_2918475
Goat Anti-mouse IgG HRP	Invitrogen	Cat# 31430; RRID: AB_228307
Goat Anti-rabbit IgG HRP	Invitrogen	Cat# 31460; RRID: AB_228341

**Biological samples**		

Human GC tissues	Shanghai East Hospital, School of Medicine, Tongji Universiy	N/A

**Chemicals, peptides, and recombinant proteins**		

A485 (p300/CBP inhibitor)	Selleck	Cat# S8740
Irinotecan Hydrochloride Trihydrate (CPT-11)	Wako	Cat# 091-06651
DMEM	Life Technologies, Gibco®	Cat# 11995-065
RPMI 1640	Life Technologies, Gibco®	Cat# 22400-089
Fetal Bovine Serum (FBS)	Life Technologies, Gibco®	Cat# A5669701
Lipofectamine 3000 Transfection Reagent	Thermo Fisher scientific	Cat# L3000075
Lipofectamine RNAiMax Transfection Reagent	Thermo Fisher scientific	Cat# 13778150
HindIII restriction enzyme	Takara Bio	Cat# 1615
BamHI restriction enzyme	Takara Bio	Cat# 1010
XhoI restriction enzyme	Takara Bio	Cat# 1635

**Critical commercial assays**		

Cell Proliferation Kit II	Roche	Cat# 11465015001
FastGene RNA Kit	Nippon Genetics	Cat# FG-81250
High Capacity cDNA Reverse Transcription Kit	Applied biosystems	Cat# 4374966
PrimeSTAR Mutagenesis Basal Kit	Takara Bio	Cat# R046A
BigDye Terminator v3.1 Cycle Sequencing Kit	Applied biosystems	Cat# 4337455
PicaGene Dual Sea Pansy Luminescence Kit	Toyo ink	Cat# 307-05581
Luna Universal One-Step RT-qPCR Kit	New England BioLabs	Cat# E3005
XTT cell Proliferation Kit II	Roche	Cat# 11465015001

**Experimental models: Cell lines**		

AGS	ATCC	Cat# CVCL0139; RRID: CVCL_0139
NUGC4	JCRB	Cat# JCRB0834; RRID: CVCL_3082

**Oligonucleotides**		

Primers for RT-qPCR, vectors construction and sequencing	See Table S1 for sequences	N/A
siRNA against KLF13 (SR309879)	Origene	Cat# SR309879
siRNA against Control (Si-NC)	Origene	Cat# SR30004

**Recombinant DNA**		

pFLAG-CMV-6c vector	Sigma-Aldrich	Cat#E2150
pGL4.10[luc2] vector	Promega	Cat# E6651
pRL-TK vector	Promega	Cat# E2241
KLF13-pFLAG expression plasmid	This paper	N/A
KLF13 mutant plasmids (K226R/227R, K226Q/227Q)	This paper	N/A
CES2 promoter-reporter plasmids (-1919, -1300, -300)	This paper	N/A
CES2 promoter mutant plasmids	This paper	N/A

**Software and algorithms**		

Graph Prism 8	GraphPad Software	https://www.graphpad.com/; RRID: SCR_002798
ImageJ	National Institutes of Health	https://imagej.nih.gov/ij/; RRID:SCR_003070
JASPAR	JAPSPAR database	http://jaspar.genereg.net/; RRID:SCR_003030
SnapGene	SnapGene	http://www.snapgene.com/;RRID:SCR_015052

### Experimental model and study participant details

#### Human gastric cancer tissues and patient cohort

A total of thirty-seven pairs of advanced gastric cancer tissue and adjacent non-tumor tissue were obtained from patients who underwent surgery at the Department of Gastrointestinal, Shanghai East Hospital, Tongji University between 2015 and 2020. This cohort included 25 males and 12 females, with a median age of 62 years (range: 45-78 years). All studies involving human subjects were approved by the Medical Research Ethics Committee of Shanghai East Hospital (Approval No: 2021−341), and written informed consent was obtained from all patients. All tumor and adjacent non-tumor tissues were processed immediately after surgical resection. Adjacent non-tumor tissue samples were procured during the same surgical procedure as the primary tumor resection. These control tissues were obtained from a region located at least 5 cm away from the tumor margin. Only pathologically confirmed non-neoplastic gastric mucosa was used as the control. Samples for RNA analysis were stored in RNAlater at -80°C, while samples for histology were formalin-fixed and paraffin-embedded (FFPE). All specimens were collected between 2015 and 2020 and were stored for a maximum of 8 years under standard conditions prior to analysis. For the analysis of treatment response, a cohort of 30 patients with pathologically confirmed, unresectable advanced or metastatic gastric cancer who received CPT-11-based chemotherapy as a second-line or later-line treatment were retrospectively included. This sub-cohort consisted of 20 males and 10 females with a median age of 64 years (range 46-75 years). Pre-treatment tumor specimens were used for gene expression analysis. No significant association was observed between patient sex and treatment response or gene expression levels in this cohort.

#### Cell lines

The human gastric cancer cell lines NUGC4 (JCRB0834) and AGS (ATCC CRL-1739) were obtained from the Japanese Collection of Research Bioresources (JCRB) and the American Type Culture Collection (ATCC), respectively. Cells were cultured in RPMI 1640 medium (Gibco) supplemented with 10% FBS (Gibco) and 1% penicillin/streptomycin at 37°C in a humidified atmosphere with 5% CO_2_. Cell lines were authenticated by STR profiling and routinely tested for mycoplasma contamination.

### Method details

#### Plasmid construction and site-directed mutagenesis

The full-length cDNA of human KLF13 was amplified from total RNA extracted from AGS cells and subcloned into the pFLAG-CMV-6c vector. Acetylation-site mutants (K226R/227R and K226Q/227Q) were generated from the wild-type plasmid using the PrimeSTAR Mutagenesis Basal Kit (Takara-Bio). The CES2 promoter region (∼1.9 kb upstream of the TSS) and its serial 5' deletion mutants (-1312 bp, -300 bp) were amplified from human genomic DNA and cloned into the pGL4.10[luc2] vector (Promega). Site-directed mutagenesis of predicted KLF13 binding sites on the -1312 bp promoter construct was also performed. All constructs were verified by Sanger sequencing. Primer sequences are listed in Table S1.

#### Transient transfection

For plasmid overexpression, cells were transfected with the indicated constructs using Lipofectamine 3000 (Invitrogen). For siRNA-mediated knockdown, cells were transfected with 20 nM of KLF13-targeting or non-targeting control siRNA duplexes (Origene) using Lipofectamine RNAiMax (Invitrogen). Experiments were typically performed 48-72 h post-transfection.

#### RNA extraction and real-time quantitative PCR (qPCR)

Total RNA was extracted using the FastGene RNA Kit (Nippon Genetics) and reverse transcribed using the High-Capacity cDNA Reverse Transcription Kit (Applied Biosystems). qPCR was performed in triplicate on a LightCycler 96 system (Roche) using the Luna Universal One-Step RT-qPCR Kit (New England BioLabs). Relative mRNA expression was calculated using the 2^-ΔΔCt^ method with GAPDH as the normalization control.

#### Western Blotting (WB)

Cells were lysed in RIPA buffer supplemented with protease and phosphatase inhibitors. Protein concentration was determined by BCA assay. Equal amounts of protein (20-30 μg) were resolved by SDS-PAGE, transferred to PVDF membranes, and probed with the primary antibodies listed in the key resources table. HRP-conjugated secondary antibodies and an ECL detection system were used for visualization. Band intensities were quantified using ImageJ software.

#### Co-immunoprecipitation (Co-IP) and acetylation assays

Cells were lysed in a non-denaturing IP lysis buffer. For endogenous acetylation assays, deacetylase inhibitors (1 μM TSA and 5 mM Nicotinamide) were added. Lysates were incubated with the indicated primary antibody or control IgG overnight at 4°C, followed by capture with Protein A/G PLUS-Agarose beads (Santa Cruz). Immunoprecipitates were washed extensively, eluted by boiling in Laemmli buffer, and analyzed by WB. CES2 Promoter Reporter Assay.

#### Luciferase reporter assay

Cells were co-transfected with a firefly luciferase reporter construct, a Renilla luciferase internal control vector (pRL-TK), and the indicated KLF13 expression plasmid (or empty vector). 48 h post-transfection, luciferase activities were measured using the Dual-Luciferase Reporter Assay System (Promega). Firefly luciferase activity was normalized to Renilla activity.

#### XTT cell viability assay

Cells were seeded in 96-well plates and treated with serially diluted CPT-11 for 48-72 h. For inhibitor studies, cells were pre-treated with A485 (3 μM) or DMSO vehicle. Cell viability was determined using the XTT Cell Proliferation Kit II (Roche), and absorbance was measured at 450 nm. Viability was expressed as a percentage of vehicle-treated controls, and IC50 values were calculated by non-linear regression using GraphPad Prism.

#### Clinical response evaluation

Objective response to CPT-11-based chemotherapy was evaluated according to the Response Evaluation Criteria in Solid Tumors (RECIST) version 1.1. The Objective Response Rate (ORR) was defined as the proportion of patients who achieved a Complete Response (CR) or Partial Response (PR).

### Quantification and statistical analysis

All quantitative data are presented as mean ± SD from at least three independent experiments. Statistical significance was determined using GraphPad Prism 8. For comparisons between two groups, a two-tailed, unpaired Student's t-test was used. For comparisons among three or more groups, one-way ANOVA with Tukey's post-hoc test was applied. Due to the limited sample size, subgroup analyses based on sex were not performed. The association between categorical clinical data was analyzed using Fisher's exact test. Pearson's correlation coefficient was used to assess the linear relationship between KLF13 and CES2 mRNA levels. A p-value of < 0.05 was considered statistically significant (∗p < 0.05).
