# Supplementary material for: KLF13-mediated CES2 upregulation via p300-dependent acetylation sensitizes gastric cancer cells to irinotecan
Source: iScience. 2025 Nov 22;28(12):114199. doi: 10.1016/j.isci.2025.114199 (PMC12721188; doi:10.1016/j.isci.2025.114199)
Supplement: Document S1. Figures S1 and S2 and Table S1 [file mmc1.pdf]

iScience, Volume *28*

Supplemental information

KLF13-mediated CES2 upregulation via p300-dependent acetylation sensitizes gastric cancer cells to irinotecan

Hai-bin Zhang, Ren-hao Hu, Ke-hui Zhang, Xi-mao Cui, and Shun Zhang

Supplement Figure 1. Validation of CES isoform expression and KLF13 gene manipulation efficiency. Related to Figure 1 and Figure 2.

(A and B) Box plots from the UALCAN portal (http://ualcan.path.uab.edu/) showing the relative mRNA expression (Transcripts Per Million, TPM) of (A) *CES1* and (B) *CES2* in normal gastric tissues (n=34) and primary gastric adenocarcinoma (STAD) tissues (n=415) from The Cancer Genome Atlas (TCGA) database. Note the difference in the y-axis scale between the two panels. Statistical significance was determined by Student's t-test and p-values are indicated.

(C) Real-time quantitative PCR (qPCR) analysis of endogenous *CES1* and *CES2* baseline mRNA expression in NUGC4 and AGS cell lines. Expression is shown relative to *CES1* expression in NUGC4 cells (set to 1).

(D) qPCR analysis of *CES1* mRNA expression in NUGC4 and AGS cells 48 h after transfection with a KLF13 overexpression (OE) plasmid or a mock vector.

(E) qPCR analysis confirming the efficiency of KLF13 overexpression. *KLF13* mRNA levels were measured in NUGC4 and AGS cells 48 h after transfection with a KLF13 OE plasmid or a mock vector.

(F) qPCR analysis confirming the efficiency of KLF13 knockdown. *KLF13* mRNA levels were measured in NUGC4 and AGS cells 72 h after transfection with KLF13-targeting siRNA (si-KLF13) or a non-targeting control siRNA (si-NC).

Data in (C-F) are presented as mean ± SD from three independent experiments. ns, not significant; $*p < 0.05$.

Supplement Figure 1

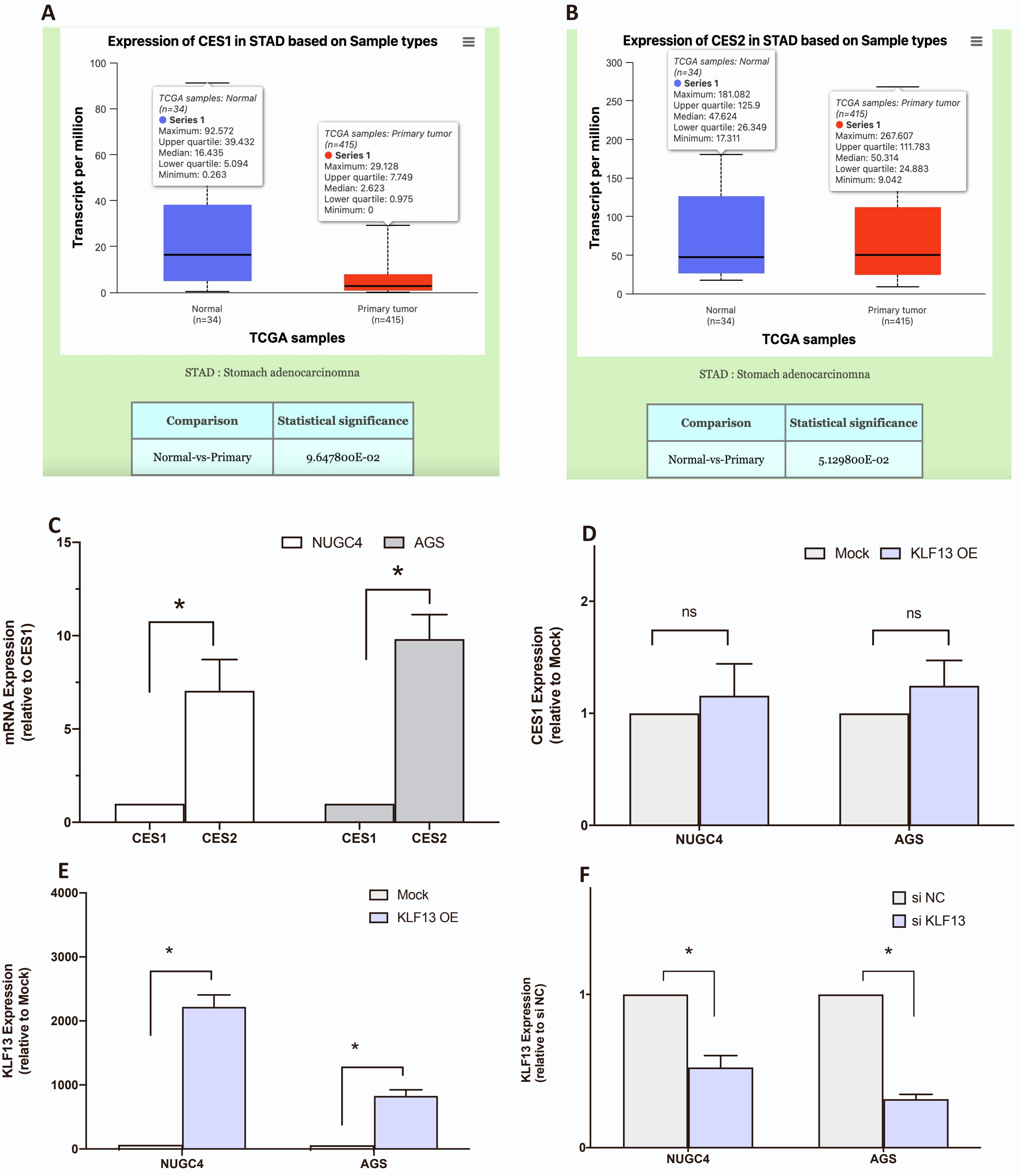

Supplement Figure 2. Expression validation of KLF13 mutant constructs. Related to Figure 5.

(A) Real-time quantitative PCR (qPCR) analysis of *KLF13* mRNA expression in NUGC4 and AGS cells 48 h after transfection with a mock vector, a wild-type KLF13 overexpression plasmid (KLF13-WT), an acetylation-deficient mutant plasmid (K226/227R), or an acetylation-mimicking mutant plasmid (K226/227Q). Data are presented as mean ± SD from three independent experiments. Statistical analysis was performed using one-way ANOVA with Tukey's post-hoc test. ns, not significant; $*p < 0.05$ (compared to mock). There was no significant difference among the three KLF13 overexpression constructs.

Supplement Figure 2

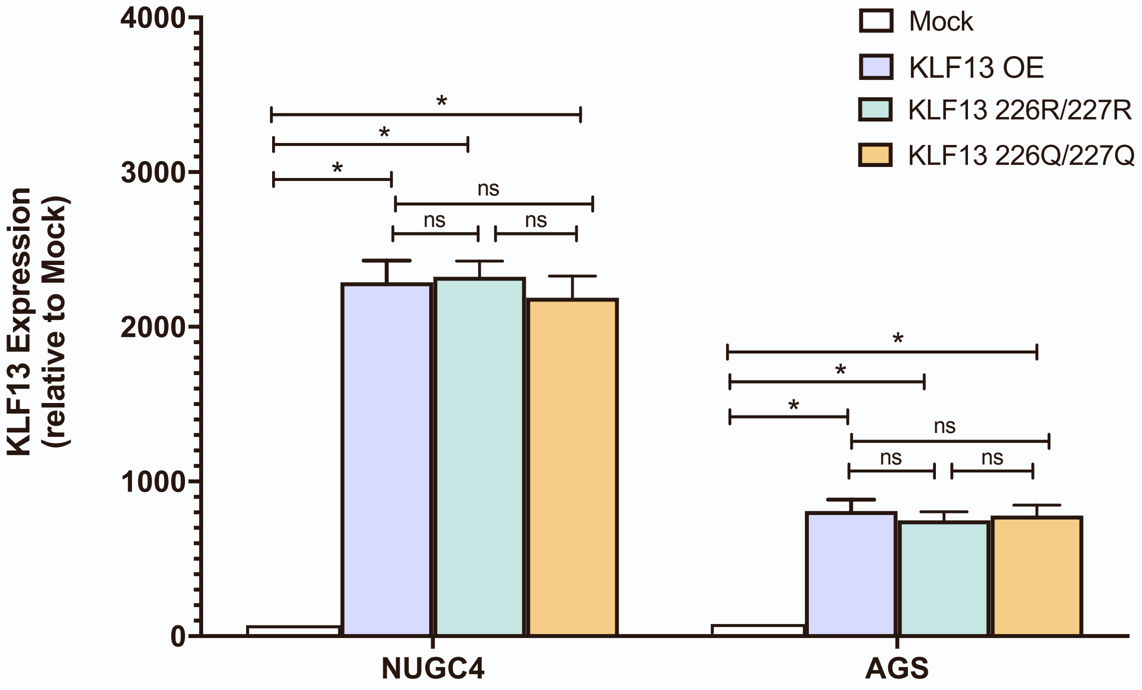

Table S1 Primers for RT qPCR, plasmid construction and sequencing

Primer	Sequence	Application
Primer CES2-Forward	GTAGCACATTTTCAGTGTTCC	Real-Time PCR
CES2-Reverse	GTAGTTGCCCCCAAAGAA	Real-Time PCR
KLF13-Forward	CGGCCTCAGACAAAGGGTC	Real-Time PCR
KLF13-Reverse	TTCCCGTAAACTTTCTCGCAG	Real-Time PCR
GAPDH-Forward	TGCCCTCAACGACC ACTTTG	Real-Time PCR
GAPDH-Reverse	CTCTTCCTCTTGTGCTCTT GCTG	Real-Time PCR
KLF13 Fwd_Hind III	AAAAAAGCTTATGGCAGCCGCCGCCTATGT	Plasmid Construction
KLF13_Rev_BamHI	AAAAGGATCC TCAGGGCGAGCTGGCCGGGC	Plasmid Construction
KLF13_K226R/K227R_Fwd	GGCGAGAGGAGGTTCAGCTGCCCCATC	Plasmid mutation
KLF13_K226R/K227R_Rev	GCTGAACCTCCTCTCGCCCGTGTGTGT	Plasmid mutation
KLF13_K226Q/K227Q_Fwd	GGCGAGCAGCAGTTCAGCTGCCCCATC	Plasmid mutation
KLF13_K226Q/K227Q_Rev	GCTGAACTGCTGCTCGCCCGTGTGTGT	Plasmid mutation
N-CMV-30	AATGTCGTAATAACCCCGCCCCGTTGACGC	Plasmid sequence
C-CMV-24	TATTAGGACAAGGCTGGTGGGCAC	Plasmid sequence
hKFL4-M	CTGCAGCTTCACCTATCCGA	Plasmid sequence
CES2-1869XhoI	AAAACTCGAGGACCTTGATGGACAGACCAA	Reporter Construction
CES2-1262XhoI	AAAACTCGAGAACCTGCTCGTCCTCCTC	Reporter Construction/Reporter sequence
CES2-250XhoI	AAAACTCGAGTATCGATCCCCCAGCGCG	Reporter Construction
CES2+50HindIII	AAAAAAGCTTGTCCAGCAGTGGATCAGTGC	Reporter Construction
CES2-45/-39-mut-Fwd	CCTACCCACATGACCTTTCCCGGCCCAAGC	Reporter mutation
CES2-45/-39-mut-Rev	AAAGGTCATGTGGGTAGGAGAGCGGGACTG	Reporter mutation
CES2-1042/-1036-mut-Fwd	AGGGACCACATGCGTTGTGGGTTCTCGGCC	Reporter mutation
CES2-1042/-1036-mut-Rev	ACAACGCATGTGGTCCCTCGCGCGACTCCG	Reporter mutation
CES2--1076/-1070mut-Fwd	GGAAGGCATGTGGGCGTGGCGACGGAGTCG	Reporter mutation
CES2-1076/-107-mut-Rev	CACGCCCACATGCCTTCCCGTTCTCCGGAA	Reporter mutation
RVprimer3	CTAGCAAAATAGGCTGTCCC	Reporter sequence
luc2St-R	CTTCTTAATGTTTTTGGCATCTTCC	Reporter sequence
